# Supplementary material for: HIVprotI: an integrated web based platform for prediction and design of HIV proteins inhibitors
Source: J Cheminform. 2018 Mar 9;10:12. doi: 10.1186/s13321-018-0266-y (PMC5845081; doi:10.1186/s13321-018-0266-y)
Supplement: Supplementary file 2 — Additional file 2. Source code of HIVProtI web server. [file 13321_2018_266_MOESM2_ESM.zip › HIVprotI_Source-code/hivprotI/weka-3-6-6/doc/weka/core/FastVector.FastVectorEnumeration.html]

FastVector.FastVectorEnumeration


---


|  |  |  |
| --- | --- | --- |
| **Overview** \| **Package** \| **Class** \| **Tree** \| **Deprecated** \| **Index** \| **Help** \| **Weka's home** | |  |
| **PREV CLASS**   **NEXT CLASS** | **FRAMES**    **NO FRAMES**     **All Classes** |  |
| SUMMARY: NESTED \| FIELD \| CONSTR \| METHOD | DETAIL: FIELD \| CONSTR \| METHOD |  |


---


## weka.core Class FastVector.FastVectorEnumeration

```
java.lang.Object
  weka.core.FastVector.FastVectorEnumeration
```

**All Implemented Interfaces:**: java.util.Enumeration, RevisionHandler

**Enclosing class:**: FastVector

---

``` public class FastVector.FastVectorEnumeration extends java.lang.Object implements java.util.Enumeration, RevisionHandler ```

Class for enumerating the vector's elements.

---

**Constructor Summary**

`FastVector.FastVectorEnumeration(FastVector vector)`
  
          Constructs an enumeration.

`FastVector.FastVectorEnumeration(FastVector vector,
int special)`
  
          Constructs an enumeration with a special element.


| **Method Summary** | |
| --- | --- |
| `java.lang.String` | `getRevision()`             Returns the revision string. |
| `boolean` | `hasMoreElements()`             Tests if there are any more elements to enumerate. |
| `java.lang.Object` | `nextElement()`             Returns the next element. |

**Methods inherited from class java.lang.Object**

`equals, getClass, hashCode, notify, notifyAll, toString, wait, wait, wait`

**Constructor Detail**

### FastVector.FastVectorEnumeration

```
public FastVector.FastVectorEnumeration(FastVector vector)
```

:   Constructs an enumeration.

    **Parameters:**: `vector` - the vector which is to be enumerated

---


### FastVector.FastVectorEnumeration

```
public FastVector.FastVectorEnumeration(FastVector vector,
                                        int special)
```

:   Constructs an enumeration with a special element.
    The special element is skipped during the enumeration.

    **Parameters:**: `vector` - the vector which is to be enumerated: `special` - the index of the special element


**Method Detail**

### hasMoreElements

```
public final boolean hasMoreElements()
```

:   Tests if there are any more elements to enumerate.

    :   **Specified by:**: `hasMoreElements` in interface `java.util.Enumeration`
    :   **Returns:**: true if there are some elements left

---


### nextElement

```
public final java.lang.Object nextElement()
```

:   Returns the next element.

    :   **Specified by:**: `nextElement` in interface `java.util.Enumeration`
    :   **Returns:**: the next element to be enumerated

---


### getRevision

```
public java.lang.String getRevision()
```

:   Returns the revision string.

    :   **Specified by:**: `getRevision` in interface `RevisionHandler`
    :   **Returns:**: the revision


---


|  |  |  |
| --- | --- | --- |
| **Overview** \| **Package** \| **Class** \| **Tree** \| **Deprecated** \| **Index** \| **Help** \| **Weka's home** | |  |
| **PREV CLASS**   **NEXT CLASS** | **FRAMES**    **NO FRAMES**     **All Classes** |  |
| SUMMARY: NESTED \| FIELD \| CONSTR \| METHOD | DETAIL: FIELD \| CONSTR \| METHOD |  |


---
